# Evidence for Microchimerism in Baboon Recipients of Pig Hearts

**DOI:** 10.3390/v15071618

**Published:** 2023-07-24

**Authors:** Hina Jhelum, Martin Bender, Bruno Reichart, Maren Mokelke, Julia Radan, Elisabeth Neumann, Ludwig Krabben, Jan-Michael Abicht, Benedikt Kaufer, Matthias Längin, Joachim Denner

**Affiliations:** 1 Institut of Virology, Free University Berlin, 14163 Berlin, Germany; hina.jhelum@fu-berlin.de (H.J.); ludwig.krabben@fu-berlin.de (L.K.); benedikt.kaufer@fu-berlin.de (B.K.); 2 Department of Anaesthesiology, University Hospital, LMU Munich, 81377 Munich, Germany; martin.bender@med.uni-muenchen.de (M.B.); jan.abicht@med.uni-muenchen.de (J.-M.A.); matthias.laengin@med.uni-muenchen.de (M.L.); 3 Transregional Collaborative Research Center 127, Walter Brendel Centre of Experimental Medicine, LMU Munich, 81377 Munich, Germany; bruno.reichart@med.uni-muenchen.de; 4 Department of Cardiac Surgery, University Hospital, LMU Munich, 81377 Munich, Germany; maren.mokelke@biontech.de (M.M.); julia.radan@med.uni-muenchen.de (J.R.); elisabeth.neumann@med.uni-muenchen.de (E.N.)

**Keywords:** xenotransplantation, porcine endogenous retroviruses, microchimerism, pig genes, SINE

## Abstract

Xenotransplantation, like allotransplantation, is usually associated with microchimerism, i.e., the presence of cells from the donor in the recipient. Microchimerism was reported in first xenotransplantation trials in humans, as well as in most preclinical trials in nonhuman primates (for review, see Denner, Viruses 2023, 15, 190). When using pigs as xenotransplantation donors, their cells contain porcine endogenous retroviruses (PERVs) in their genome. This makes it difficult to discriminate between microchimerism and PERV infection of the recipient. Here, we demonstrate the appropriate virological methods to be used for the identification of microchimerism, first by screening for porcine cellular genes, and then how to detect infection of the host. Using porcine short interspersed nuclear sequences (SINEs), which have hundreds of thousands of copies in the pig genome, significantly increased the sensitivity of the screening for pig cells. Second, absence of PERV RNA demonstrated an absence of viral genomic RNA or expression as mRNA. Lastly, absence of antibodies against PERV proteins conclusively demonstrated an absence of a PERV infection. When applying these methods for analyzing baboons after pig heart transplantation, microchimerism could be demonstrated and infection excluded in all animals. These methods can be used in future clinical trials.

## 1. Introduction

Microchimerism is a condition in which small numbers of cells from one individual are present in another, genetically different, individual’s body [1,2]. Microchimerism is quite common. The most common form is fetomaternal microchimerism when cells from the developing fetus cross the placenta and enter the mother’s bloodstream, or when a mother’s cells cross into the developing fetus [1,2,3,4,5]. It is important to note that the cells from the fetus or from the mother may persist in the other individual for decades [3,4]. Notably, fetal microchimeric cells were reported to show progenitor cell and stem-cell phenotypes (for a review, see [6]). Microchimerism can have both positive and negative effects. For example, fetal cells in the mother’s body may play a role in tissue repair and immune function, while also potentially contributing to autoimmune diseases [3,4].

Microchimerism was also described in allotransplantation, showing the presence of cells from organ transplant donors in the corresponding recipients, even after the organ was rejected [7,8,9]. Microchimerism was observed in recipients after kidney allotransplantation, as well as in liver and lung recipients [10]. Similar to the situation in pregnancy, donor cells in transplant recipients can cause graft-versus-host disease; on the other hand, they may contribute to a survival of the transplant with reduced pharmaceutical immunosuppression or even immunological tolerance [11]. Microchimerism can also occur following allogeneic blood transfusion in patients, where donor cells have been detected decades after transfusion [12,13], as well as after stem-cell treatment [14]. Atypical lymphocytes appeared in the circulation of a large proportion of patients during the first week following a blood transfusion, and they had spontaneously dividing mononuclear cells of the recipient or host karyotype [15]. Overall, microchimerism is an area of ongoing research and it is important to fully understand the implications and effects of this phenomenon on the human body.

Microchimerism was not only detected in recipients of allotransplants, but also in recipients of xenotransplants, both in nonhuman primates in preclinical trials [16] and in humans in clinical trials such as extracorporeal splenic perfusion [17], transplanting encapsulated pig islet cells into diabetic patients [18], or others (for review see [1]). For example, in a preclinical study of orthotopic pig heart transplantation, PERV sequences were detected in the blood samples in all eight transplanted baboons, and it was assumed that this was due to circulating cellular DNA released from dead transplant cells or from circulating pig cells, e.g., microchimerism [16]. Most importantly, Western blot analysis showed no anti-PERV antibodies in the serum of the baboons, clearly demonstrating absence of an infection [16].

Microchimerism is easier to detect in xenotransplantation than in allotransplantation. In allotransplantation, the absence or presence of the Y chromosome or differences in the HLA molecules were used for differentiation [1], molecules of a different species can easily be detected. PCR methods have been used to detect pig-specific centromeric or mitochondrial sequences which are present in the pig genome in many copies [17]. In the analysis of patients who received pig islet cells, COX, a mitochondrial cytochrome oxidase gene, which is present in most cell types in hundreds to thousand copies, was used [19]. Positive results for COX were observed in some samples indicating that porcine material was transiently detectable in the bloodstream of several human transplant recipients at various timepoints post transplantation [19]. Since there are up to 60 PERV copies in a pig cell, the detection by PCR using highly conserved PERV polymerase (pol) primers is very sensitive [20]. However, this method does not allow discriminating between microchimerism and potential PERV infection. A more sensitive approach is the use of short interspersed nuclear elements (SINEs) which are present in pig cells with a copy number of more than 100,000. SINEs are a group of interspersed repetitive sequences found in mammalian genomes, the non-LTR (long terminal repeats) retrotransposons. Non-LTR retrotransposons are divided into two groups primarily by their size: SINEs (in humans, Alu) and long interspersed nuclear sequences (LINEs; in humans, L1). PRE-1 is the major SINE of the pig genome [21]. The 233 bp PRE-1 sequence contains an RNA polymerase III split promoter (16–27 bp and 58–69 bp), as well as two short direct repeats (91–124 bp and 138–170 bp) (Supplementary Figure S1). The 3′ termini of the elements consist of a poly A tail of variable length [22]. It was estimated that there are 100,000 to 1,000,000 copies per haploid genome [21,22]. Although none of these elements contains an open reading frame, they are transcribed in some pig tissues [21]. PRE-1 sequences are unevenly distributed along the chromosomes as in the case of the human and mouse SINEs. However, there is a difference; PRE-1 is localized on centromeric regions, whereas human and mouse SINEs are not [23]. Hypermethylation of the repetitive region PRE-1 was found to be associated with defective development and early abortion of cloned pigs [24]. Since the sequence is specific for pigs [21,25], it can be used to detect pig DNA and consequently pig cells with a thousand-fold higher sensitivity compared with cellular genes such as pGAPDH. This method was suitable for the detection of microcontamination of feed with animal materials [22] and commercially purchased meat products [25].

Here, we analyzed different organs from four baboons, which received a heart from triple genetically modified pigs (GGTA1-KO, hCD46, and hTBM) [26] for the presence of PERV sequences, porcine GAPDH, and SINE sequences. Furthermore, a RT real-time PCR was performed to detect expression of PERV as RNA. We also screened the explanted hearts for the presence of baboon cells.

## 2. Materials and Methods

### 2.1. Tissues from Transplanted Baboons

Tissue samples of skin, kidney, spleen, lung, aorta, and peripheral blood mononuclear cells were taken from the transplanted baboons after euthanasia; in addition, tissue samples of the pericardium and the left ventricle were taken from the explanted pig heart All samples were frozen at −80 °C. Four animals were analyzed (Table 1). Animal A was new, whereas animals B, C, and D had been included in the study described in [16], baboon B corresponds to animal O, baboon C corresponds to animal Q, and baboon D corresponds to animal K. All four baboons received an orthotopic heart transplantation from a pig which was triple genetically modified: GGTA1-KO (knockout of the porcine GGTA1 gene which encodes for the α1,3-galactosyltransferase that synthesizes the Gal epitopes), hCD46 (expression of the human CD46, also called membrane cofactor protein, MCP), and hTBM (expression of the human thrombomodulin) [26].

All animals received basic immunosuppression, as described previously [26,27]; induction therapy included anti-CD20 antibody, anti-thymocyte-globulin, and an anti-CD40 or an anti-CD40L antibody. During maintenance therapy, methylprednisolone was reduced gradually, whereas mycophenolate mofetil and the anti-CD40 or anti-CD40L treatment remained constant.

### 2.2. DNA and RNA Isolation

DNA and RNA were isolated from the tissues according to manufacturer’s instructions using the DNeasy Blood and Tissue kit and RNeasy kit (Qiagen, Hilden, Germany), respectively. DNA and RNA concentrations were determined using NanoDrop ND-1000 (Thermo Fisher Scientific Inc., Worcester, MA, USA) or Invitrogen Qubit 4 Fluorometer (Waltham, MA, USA).

### 2.3. PCR Methods

The PRE-1 PCR was performed using the primers described by [25] (Table 2) and the following conditions: 2 ng of DNA template, 1× PCR buffer I containing MgCl_2_, 0.2 mM dNTPs, and 1 unit of AmpliTaq DNA polymerase (Applied Biosystems, Inc., Waltham, MA, USA). Each sample was subjected to an initial denaturation of 1 min at 95 °C, followed by 30 amplification cycles (95 °C for 30 s, 60 °C for 1 min, and 72 °C for 1 min) and a final extension at 72 °C for 5 min to get an amplicon of 134 bp size. The PRE-1 PCR assay had a linear quantitation range of 10–0.00001 ng (0.01 pg), or 10^7^, as shown by [25].

### 2.4. Real-Time PCR Methods

PRE-1 real-time PCR was carried out using the primers and the probe described by [25], with the following conditions in a 20 µL reaction volume: 100 ng of DNA applying a temperature–time profile that consisted of initial denaturation of 5 min at 95 °C, followed by 45 amplification cycles of denaturation at 95 °C for 15 s, annealing at 60 °C for 30 s, and extension at 72 °C for 30 s.

The PERV pol real-time PCR was performed using primers and a probe described by [28] (Table 2), and the annealing temperature was changed to 62 °C to achieve a sensitivity of 10 copies. This PCR was run as a duplex PCR with primers and probes for the porcine glyceraldehyde 3-phosphate dehydrogenase (pGAPDH) and human GAPDH (hGAPDH), which also recognizes the baboon GAPDH (Table 2).

All experiments were performed with the SensiFAST Probe No-ROX kit (Meridian Bioscience, Cincinnati, OH, USA) at the qPCR cycler qTOWER3 G (Analytik Jena, Jena, Germany).

### 2.5. Testing for PCMV/PRV; PCV3, CR Methods

PCR testing for PCMV/PRV, HEV, and PLHV-1/2 was performed as described in [16], for PCV3 as described in [29]. Virus testing was performed in order to analyze whether virus infections may influence the extend of microchimerism.

## 3. Results

### 3.1. Improvement of the Detection Methods

In order to detect PERV sequences, a real-time PCR was performed with primers and a probe binding to a highly conserved region in the PERV polymerase gene (pol) [28]. The conditions were modified in such a way (see Section 2) that 10 copies were detected (Supplementary Table S1). The real-time PCR was performed as a duplex PCR detecting in parallel porcine glyceraldehyde 3-phosphate dehydrogenase (pGAPDH). The positive control was a gene block containing the PERV pol sequence between both primer binding sites, as well as the target sequences of the real-time PCRs detecting pGAPDH and human GAPDH (hGAPDH) [30]. The sequences of the primer binding sites and probe in hGAPDH were identical to the primer binding sites and probe of baboon GAPDH.

**Figure 1:**
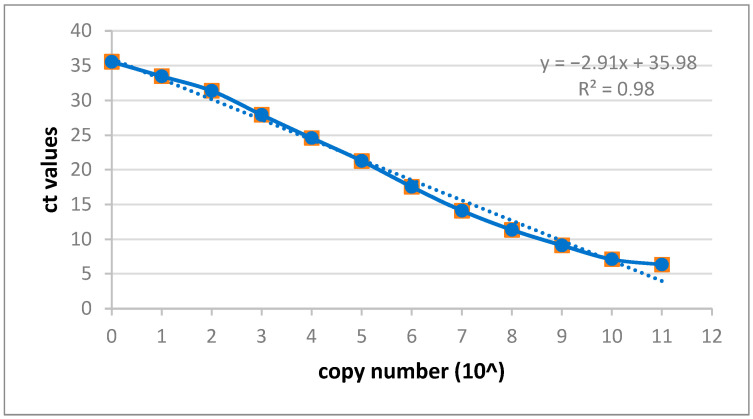
Standard curve of the real-time PCR using primers and probes for the PRE-1 sequence.

Although the conventional PCR detecting pig SINE sequences (PRE-1) worked well (not shown), a new real-time PCR was developed with the same primers and in a specific probe (Table 1). A dilution of the amplicon product of the PRE-1 PCR was used to obtain a standard curve for future determinations of the copy number (Figure 1).

The species specificity of the primers and probes detecting porcine GAPDH, human/baboon GAPDH, and SINE was shown. The primers and probes specific for SINE did not detect repetitive sequences in non-transplanted baboon and human DNA.

**Table 2:** Primers and probes used for the PCRs and real-time PCRs.

Gen	Primer/Probe	Sequence	Location (Nucleotid Number)	Accession Number	Reference
PRE-1	PRE-1 fwd	5‘ GACTAGGAACCATGAGGTTGCG 3′	37–58	GenBank Y00104	Walker et al., 2003 [25]
	PRE-1 rev	5′ AGCCTACACCACAGCCACAG 3′	61–85		
	PRE-1 probe	5′ FAM-TTTGATCCCTGGCCTTGCTCAGTGG-BHQ1 3′	151–170		
pGAPDH	pGAPDH fwd	GAT CGA GTT GGG GCT GTG ACT	1083–1104	GenBank NM_001206359.1	Duvigneau et al., 2005 [31]
	pGAPDH rev	ACA TGG CCT CCA AGG AGT AAG A	1188–1168		
	pGAPDH probe	HEX-CCA CCA ACC CCA GCA AGA GCA CGC-BHQ	1114–1137		
hGAPDH	hGAPDH fwd	GGCGATGCTGGCGCTGAGTAC	3568–3587	GenBank AF261085	Behrendt et al., 2009 [32]
	hGAPDH rev	TGGTCCACACCCATGACGA	3803–3783		
	hGAPDH probe	HEX-CTTCACCACCATGGAGAAGGCTGGG-BHQ1	3655–3678		
PERV pol	PERV pol fwd	CGACTG CCCCAAGGG TTC AA	3568–3587	GenBank HM159246	Yang et al., 2015 [28]
	PERV pol rev	TCTCTCCTG CAA ATC TGG GCC	3803–3783		
	PERV pol probe	6FAM-CACGTACTG GAG GAG GGTCAC CTG -BHQ1	3678–3655		

### 3.2. Detection of PERV Sequences in Baboon Tissues after Transplantation

**Figure 2:**
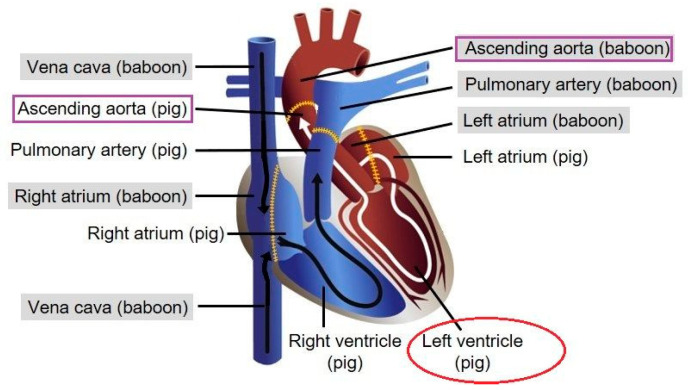
Schematic presentation of the transplanted pig heart in the blood circulation of the baboon. Dotted lines indicate the contact between pig and baboon tissues. Tissue samples from the left ventricle (red circle) and both parts of the aorta (lilac boxes) were analyzed here.

A modified and highly sensitive PERV pol real-time PCR (see above) was used to confirm previous data that PERV sequences can be detected in the organs of baboons after transplantation of pig hearts [16]. When tissues of baboon A were analyzed using this real-time PCR, PERV sequences were found in all organs with exception of the liver, with slightly different ct values, indicating different numbers of PERV sequences in different tissues (Table 3). The highest number of PERV sequences was found in the aorta. The pericardium was found to be a mixture of pig and baboon tissue, because nearly identical ct values of porcine GAPDH and baboon GAPDH were found. The left ventricle was mainly pig tissue (Figure 2), indicated by the high number of pGAPDH and PERV sequences, but there were also baboon cells present as detected by the human/baboon GAPDH PCR (see below). It is important to note that baboon A was negative for PCMV/PRV and PCV3 (Table 3).

PERV sequences were also found in tissues of the three other baboons (Table 4). In the case of baboon B, PERV sequences were found in skin, liver, and lung but not in kidney and spleen; in baboon C, they were only in liver and lung; in baboon D, they were found only in the skin. The highest number of PERV sequences was found in the aorta. This indicated that, in the baboon aorta, many pig cells accumulated, or that the analyzed sample represented the site of anastomosis, i.e., where both porcine and baboon aorta were sutured together. The sample from the pericardium also presented a mixture of porcine and baboon tissues. Baboon B was negative for PCMV/PRV, HEV, and PLHV-1, -2-, and -3 [16], but PCV3-positive [29]. Baboon C was positive for PCMV/PRV and PCV3. Baboon D was PCMV/PRV-negative, but positive for PLHV-1/2 [16,29]. We have no evidence that the transmission of these viruses influenced the dissemination of pig cells.

### 3.3. Evidence for Microchimerism

Since we proposed, on the basis of previous results [16], that the PERV sequences found in the baboon tissues were due to the presence of pig cells in the baboon tissues, e.g., due to microchimerism, a duplex real-time PCR was performed to detect a porcine cellular gene, pGAPDH. In none of the tissues of baboon A was pGAPDH detected with exception of the aorta, indicating the presence of a high percentage of pig cells (Table 2). This result was in agreement with the result of the PERVpol-specific real-time PCR, which also showed a high number of PERV sequences in the aorta. The same was observed in the other three baboons (Table 3).

The presence of PERV sequences in the tissues of the transplanted baboons indicated either infection of the recipient animal or the presence of pig cells, e.g., microchimerism. In the case of microchimerism, the detection of PERV sequences in comparison with the results with porcine GAPDH can be explained by the higher copy number of PERV (up to 60) in the pig cell genome [33], whereas pGAPDH has only two copies.

In order to answer the question of whether it is an infection or microchimerism, another pig-specific marker was used, which has a very high copy number in the pig genome compared with pGAPDH. SINE sequences were well suited for this purpose. SINE sequences were found in all the organs of baboon A (Table 3). As expected, the number of SINE sequences was very high (ct 6.54) in the left ventricle, which is pig tissue (Figure 2). The number of SINE sequences was also high in the aorta, confirming the results with PERV and pGAPDH that numerous pig cells accumulated in this vessel, or that the analyzed sample represented the site of anastomosis. Similar results were obtained in the other three animals (Table 3).

### 3.4. Baboon Cells in the Explanted Pig Heart

To analyze whether baboon cells can be found in the transplanted pig heart, a real-time PCR detecting baboon/human GAPDH was performed. Indeed, baboon cells were found in high quantities in the left ventricle of the explanted pig heart from baboon A (Table 2).

### 3.5. Absence of PERV Expression

To analyze whether PERV was expressed as RNA in the baboon tissues with the highest prevalence of pig cells, RNA was isolated from the kidney, lung, and spleen of baboon A, and an RT real-time PCR was performed using PERVpol primers and the probe. In none of these tissues was expression of PERV at the level of mRNA or genomic RNA observed (Table 5).

## 4. Discussion

**Figure 3:**
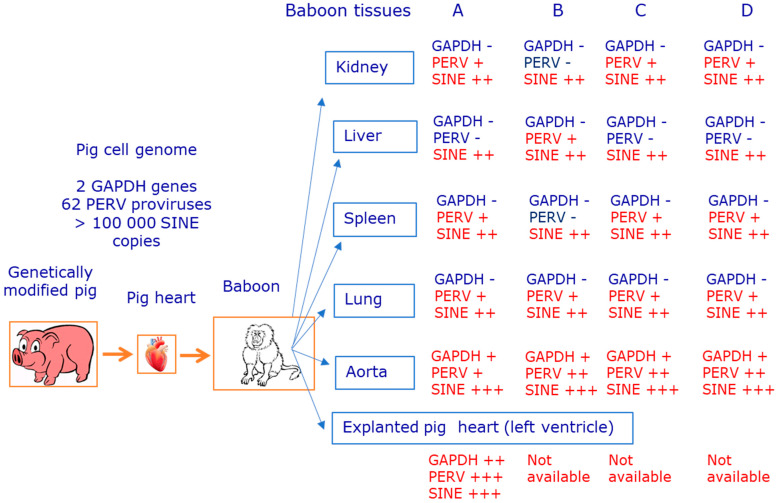
Summary of the detection of pig genes in different tissues of the transplanted baboon. −, negative, +, positive, ++, very positive, +++ positive with very low ct value. GAPDH, glyceraldehyde 3-phosphate dehydrogenase; PERV, porcine endogenous retrovirus; SINE, short interspersed nuclear elements.

Here we demonstrated that, after orthotopic transplantation of pig hearts into baboons, pig cells were found in nearly all of the analyzed organs of the recipients, which is called microchimerism (Figure 3). We developed a highly sensitive method using pig SINE sequences able to detect pig cells, and we showed that the transplanted baboons were not infected with PERV.

We demonstrated that xenotransplantation, like allotransplantation, is associated with microchimerism (Table 3 and Table 4). Furthermore, using several new and more sensitive methods, we confirmed previous results showing pig cells in transplanted baboons [16]. The number of pig cells depended on the organ and the animal, and it was relatively low. This was shown by the fact that the pig cells could not be detected using a PCR detecting pig genes such as porcine GAPDH, which are present only twice in the pig genome. A higher sensitivity of detection of pig cells was achieved using a PCR detecting PERV sequences, which are present up to 60 times in the pig genome [33] and using SINE sequences which are present more than 100,000 times in the pig genome (Table 3 and Table 4). We also showed that the pig cells were alive because we detected mRNA of porcine GAPDH (Table 5). The presence of disseminated pig cells in organs of transplanted baboons was also supported by previous studies showing disseminated cells expressing PCMV/PRV in different organs of the baboons [34]. Since PCMV/PRV was shown not to infect human cells [35], it is likely that PCMV/PRV also does not infect other primate cells including baboon cells. Therefore, it is very likely that the cells expressing PCMV/PRV, as detected by immunohistochemistry [34], in the organs of the baboons are pig cells expressing viral proteins.

The highest number of PERV sequences was found in the aorta, indicating settlement of pig cells or that the analyzed tissue sample represented the site of anastomosis. The baboon aorta is connected with the pig aorta, and the entire bloodstream comes from the pig part of the aorta to the baboon part of the aorta allowing settlement of pig cells (Figure 2).

The material collected as pericardium was not well defined. According to the ct values of pGAPDH and hGAPDH, it represents a mixture of pig and baboon tissue. In contrast, the left ventricle was mainly pig tissue, as demonstrated by the extremely high number of PERV and pGAPDH sequences. Nevertheless, there were also baboon cells present in the left ventricle, as shown by the detection of hGAPDH (Table 3). Whether this is due to baboon blood cells circulating in the pig heart or whether there are also settled baboon endothelial and other cells is unknown.

Using a real-time PCR for SINE sequences in order to detect pig cells in the transplanted baboons, an extremely sensitive method for the detection of pig cells in recipients was created. This method will also be useful when screening for pig cells in human patients after xenotransplantation. In this case, the human repetitive Alu sequences should be selected. This will also be useful for the discrimination between PERV infection and microchimerism in human patients.

However, the presence of pig genes, especially of SINE sequences, does not automatically mean that there is no infection of recipient cells, especially if only a very few recipient’s cells are infected. A strategy that better discriminates between PERV infection and microchimerism is to study insertion of the virus into the cellular DNA and to determine whether the virus is integrated in pig DNA (pig cells, microchimerism) or in baboon DNA (infection). Such studies were performed in one preclinical trial; after transplantation of pig kidneys into rhesus macaques, PERV sequences were detected in the bladder of the animals [36]. The authors could demonstrate that PERVs originated from porcine donor cells rather than an integrated provirus in the monkey chromosome. To determine PERV insertion into chromosomes, inverse PCR using PERV long terminal repeat (LTR) region-specific primers was conducted. The presence of pig cells in the monkey bladder after renal xenotransplantation was also demonstrated using specific porcine mitochondrial DNA gene PCR [36]. However, assuming that the integration into baboon DNA, e.g., infection, is a very rare event, the number of sequenced amplicons of the inverse PCR was much too low to exclude infection in some cells. This is the general disadvantage of this method.

On the basis of these results, the best method to detect a real PERV infection is to demonstrate antibodies against PERV. The detection of antibodies is the easiest way to detect retrovirus infection, and it is used for the diagnosis of human immunodeficiency viruses 1 and 2 (HIV-1, -2), human T-cell lymphotropic viruses I and II (HTLV-I, -II) [37], feline immunodeficiency virus (FIV) [38], bovine leukemia virus (BLV) [39,40], and small ruminant lentiviruses (SRLVs) [41]. Immunological assays such as Western blot assay or ELISA are easy to perform, and the sera required for testing can be obtained easily. In the past, numerous assays have been developed to detect PERV-specific antibodies, mainly Western blot assays and ELISAs using purified virus, recombinant viral proteins, or peptides [42,43,44]. Using these tests, in all cases, no antibodies have been detected in animals and humans who had received pig cells or organs, and in animals inoculated with high doses of PERV with and without pharmaceutical immunosuppression (for a review, see [45,46]), indicating that, until now, not a single PERV infection had been observed in vivo. The only exception was a limited PERV infection without evidence of replication but low antibody production in guinea pigs [47]. The argument that detection of antibodies may be hampered by the fact that the recipients are immunosuppressed can be refuted by numerous publications showing that HIV-1-infected individuals produce a strong antibody response against the virus [37], and by the fact that patients who received an allotransplant produced antibodies when vaccinated despite transplantation-associated immunosuppression [48,49,50,51].

Most importantly, the absence of PERV mRNA and genomic RNA in the cells in the baboon tissues (Table 4) makes it unlikely that viral protein and viral particles will be produced in the pig cells. Consequently, no antiviral antibodies will be produced in the baboon. This was demonstrated by negative Western blot assays of the baboons analyzed here in the previous study [16].

There is another important outcome from this study. We recently proposed to monitor xenotransplant tissue damage and rejection by the detection of cell-free pig DNA using integrated PERV sequences [20]. This suggestion was based on the finding that free extracellular DNA is a good marker of rejection in allotransplantation, and that the use of PERV sequences instead of pig cellular genes makes the method ~60 times more sensitive. On the basis of the results found here with the SINE sequences, we propose now to use SINE sequences as a much more powerful approach due to the high copy number in the pig genome. This will enormously increase the sensitivity when screening for free extracellular DNA as marker of transplant rejection.

## 5. Conclusions

Pig cells were detected in different tissues of baboons after transplantation of a pig heart. The highly sensitive detection method using primers and a probe for pig SINE is the most effective approach to detect pig cells and to exclude infection of the host. The absence of PERV-specific genomic and mRNA and the absence of PERV-specific antibodies finally excluded infection.

## Figures and Tables

**Table 1:** Baboon recipients and details of the pig heart transplantation.

Animal Number	Animal Recipient ID	Donor Genetics	Time of Sampling [POD]	Blood Group of the Donor	Blood Group of the Recipient	Study Design
A	17475	GT-KO/hCD46/hTM	195	0	AB	oHTx
B	17493	GT-KO/hCD46/hTM	194	0	B	oHTx
C	17492	GT-KO/hCD46/hTM	26	0	B	oHTx
D	17769	GT-KO/hCD46/hTM	50	0	B	oHTx

GT-KO: α1,3-galactosyltransferase homozygous knockout; hCD46: hemizygous transgenic for human CD46; hTM: hemizygous transgenic for human thrombomodulin; POD: postoperative day after xenotransplantation; oHTx: orthotopic thoracic heart transplantation.

**Table 3:** Results of the PCR testing of baboon A (green, mainly baboon; lilac, mainly pig).

Tissue/ Real-Time PCR	Ct Values					
	PCMV/PRV	PCV3	PERV Pol	pGAPDH	PRE-1	hGAPDH
Skin	N. d.	N.d.	29. 81	N.d.	22.51	20.00
Kidney	N.d.	N.d.	31.72	N.d.	24.65	18.23
Spleen	N.d.	N.d.	32.05	N.d.	19.55	17.2
Liver	N.d.	N.d.	N.d.	N.d.	20.64	17.74
Lung	N.d.	N.d.	32.47	N.d.	18.72	18.07
Aorta	N.d.	N.d.	21.91	27.49	16.4	20.89
Pericardium	N.d.	N.d.	22.83	25.69	Not available	20.22
Left ventricle	N.d.	N.d.	14.17	17.76	6.54	18.68

N.d., not detected.

**Table 4:** Detection of PERV pol, porcine GAPDH, and baboon GAPDH in baboon tissues and explanted pig tissues after the end of xenotransplantation by real-time PCR (green, mainly baboon; lilac, mainly pig). Although material from the left ventricle of the other baboons was not available, the result would be the same as in the left ventricle from baboon A.

Baboon	B				C				D			
Tissue/Real-Time PCR	Ct Values											
	PERV Pol	pGAPDH	PRE-1	hGAPDH	PERV Pol	pGAPDH	PRE-1	hGAPDH	PERV Pol	pGAPDH	PRE-1	hGAPDH
Kidney	N.d.	N.d.	19.57	18.02	N.d.	N.d.	22.25	17.89	N.d.	N.d.	22.36	19.16
Spleen	N.d.	N.d.	20.73	18.34	N.d.	N.d.	19.93	18.53	N.d.	N.d.	21.84	19.16
Liver	27.76	N.d.	20.33	20.97	32.54	N.d.	20.06	18.94	N.d.	N.d.	22.34	19.88
Lung	27.49	N.d.	17.51	17.56	32.81	N.d.	19.86	18.92	N.d.	N.d.	20.25	18.83
Aorta	24.62	28.65	15.07	18.29	N.d.	N.d.	23.17	19.30	N.d.	N.d.	24.23	20.51
Pericardium	25.40	30.16	16.47	19.36	23.29	26.85	14.11	19.23	25.16	28.9	16.13	23.23

N.d., not detected.

**Table 5:** Screening for expression of PERV pol, and baboon GAPDH in different organs of the transplanted baboon A using real-time RT-PCR (green background indicates: mainly baboon tissue).

Tissue/ Real-Time PCR	Ct Values	
	PERV Pol	hGAPDH
Kidney	N. d.	23.54
Lung	N.d.	25.24
Spleen	N.d	21.36

N.d., not detected.

## Data Availability

All data supporting reported results can be found in this publication and in the Supplementary Materials.

## Supplementary Materials

The following supporting information can be downloaded at https://www.mdpi.com/article/10.3390/v15071618/s1: Figure S1. Sequence and functional domains of PRE-1 (nucleotides 1–233) according to Singer et al. [21]. Forward and reverse primers and probes according to Walker et al. [25] are indicated, and the differences in the sequence are shown in green. The sequences of the RNA polymerase III split promotor (nt 16–27 and nt 58–69) and the short direct repeats (nt 91–124 and nt 138–170) as postulated by [18] are underlined; Table S1. Determination of the effective annealing temperature and sensitivity of the PCR detecting SINE sequences (PER-1) in the pig genome.
