# Supplementary material for: Evidence for Microchimerism in Baboon Recipients of Pig Hearts
Source: Viruses. 2023 Jul 24;15(7):1618. doi: 10.3390/v15071618 (PMC10385208; doi:10.3390/v15071618)
Supplement: Supplementary file 1 [file viruses-15-01618-s001.zip › viruses-2486897-supplementary.pdf]

Table S1. Determination of the effective annealing temperature and sensitivity of the PCR detecting PERVpol sequences using a gene block

Annealing Temperature<br>58 Degrees gBlock III Dilutions		60 Degrees	62 Degrees
10^0	N.d.	N.d.	N.d.
10^1	N.d.	N.d.	37.03
10^2	36.46	33.58	36.07
10^3	31.36	30.67	29.89
10^4	24.19	24.61	23.54
10^5	19.27	19.35	19.69

N.D., not detected

Supplementary Figure 1

```
1        10        20        30        40        50       60         70

AGGGAGTTCCCATCGTGGCTCAGTGGTAACGAACCTGACTAGTATCCATGAGGATGCAGATTCAATCCCT
                                    GACTAGGAACCATGAGGTTGCG  TTTGATCCCT

71      80        90        100       110       120       130       140

GGCCTTNCTCAGTCGGTTAAGGATCCNGCATTGCTGTGAGNTGTGGTGTAGGTCNCAGATGCGGCTCGGA
GGCCTTGCTCAGTGG

141     150       160       170       180       190       200       210

TCCCGTGTTGCTGTGGCTGTGGTGTAGGCCGGCAGCTACAGCTCCGATTNGACCCCTAGCCTGGGAACCT
          GACACCGACACCACATCCGA

211     220       230

CCATATGCCGCGGGTGNGGCCCT
```

Forward Primer GACTAGGAACCATGAGGTTGCG

Reverse Primer AGCCTACACCACAGCCACAG

Probe TTTGATCCCTGGCCTTGCTCAGTGG
